# The serum IL-17A levels in patients with traumatic bowel rupture post-surgery and its predictive value for patient prognosis

**DOI:** 10.1515/med-2024-1135

**Published:** 2025-03-05

**Authors:** Peng Zhou, Jie Yu, Bingmei Yan

**Affiliations:** Hernia and Abdominal Wall Surgery, Nursing Department, Ruian People’s Hospital, Ruian, Wenzhou, 325200, China; Gastrointestinal Surgery, Nursing Department, Ruian People’s Hospital, Ruian, Wenzhou, 325200, China; Operating Room, Ruian People’s Hospital, No.108, Wansong Road, Dongzhen Community, Yuhai Street, Ruian, Wenzhou, Zhejiang, 325200, China

**Keywords:** abdominal trauma, traumatic bowel rupture, IL-17A, cytokines, prognosis

## Abstract

**Objective:**

This study aimed to investigate the serum levels of interleukin (IL)-17A in patients with traumatic bowel rupture and its clinical significance, particularly its correlation with inflammatory cytokines, preoperative severity, and postoperative prognosis.

**Methods:**

A total of 104 patients with traumatic bowel rupture admitted to Ruian People’s Hospital between February 2021 and June 2024 were included in this prospective observational study. All patients underwent standard surgical treatment for traumatic bowel rupture at our hospital. Serum levels of IL-17A, IL-6, tumor necrosis factor-alpha, and C-reactive protein were measured at various time points using enzyme-linked immunosorbent assay. Clinical data and demographics were collected. Patients were followed up for 3 months post-discharge.

**Results:**

The study found significantly higher levels of IL-17A and IL-6 in patients with an injury severity score (ISS) of ≥16 compared to those with an ISS of <16 (*p* < 0.05). Serum IL-17A levels were particularly elevated in patients with poor prognosis (*p* < 0.05). Of particular importance, receiver operating characteristic curve analysis demonstrated that serum IL-17A levels at 72 h post-surgery had predictive value for poor prognosis, with an area under the curve of 0.773, a cutoff value of 41.75 pg/mL, a sensitivity of 62.2%, and a specificity of 83.6%. Logistic regression analysis identified elevated IL-17 levels at 72 h post-surgery as a significant risk factor for poor prognosis (odds ratio = 1.273, 95% confidence interval: 1.115–1.453, *p* < 0.001).

**Conclusion:**

In summary, our study highlights the significant potential of serum IL-17A as a biomarker for predicting poor prognosis in patients with traumatic bowel rupture post-surgery, suggesting its utility in clinical assessment and potential as a therapeutic target.

## 1 Introduction

Trauma has become the leading cause of death for individuals under the age of 45 years and the fourth leading cause of death across all age groups, imposing a significant burden on healthcare systems and the economy [1,2]. Abdominal trauma accounts for approximately 9–15% of all trauma cases and is one of the most severe types, requiring special management strategies [3,4,5,6]. Intestinal rupture is a common consequence of abdominal trauma, second only to splenic injury [7].

Traffic accidents are the most common cause of traumatic bowel rupture [8]. The condition results in the leakage of a large number of intestinal bacteria into the abdominal cavity, complicating aseptic management and increasing the risk of abdominal infections [9]. Studies have shown that the incidence of complications and mortality in patients with bowel rupture post-surgery can reach up to 20% [10]. Early activation of the inflammatory response in bowel rupture patients leads to the release of numerous pro-inflammatory factors. Delayed identification of the condition can trigger a cascade of inflammatory responses, accelerating damage to related organs and resulting in severe complications such as peritonitis, sepsis, and septicemia, posing a significant threat to patients’ lives [10,11]. Given the high risk of infection in bowel rupture patients, early recognition of the condition’s severity and identification of patients likely to develop severe complications are crucial for timely intervention. In addition to traditional diagnostic methods such as abdominal exploration and computed tomography (CT) scans, the clinical significance of biomarkers in the diagnosis and prognosis of trauma patients has gained widespread attention in recent years [12,13,14].

Interleukin (IL)-17A, the most studied member of the IL-17 cytokine family [15], is primarily produced by T cells [16]. It has been shown to play a crucial role in host defense against various microbial pathogens and tissue inflammation [17]. IL-17 can induce the production of a range of other pro-inflammatory mediators, including chemokines, cytokines, and metalloproteinases from epithelial cells, endothelial cells, and fibroblasts [18,19]. Increasing evidence suggests that IL-17 not only plays a significant pro-inflammatory role in autoimmune diseases but also has important context- and tissue-dependent roles in maintaining health during trauma, physiological stress, and infection responses [20,21,22]. A clinical study by Frangen et al. found elevated IL-17 expression in patients with severe trauma, particularly those with thoracic and abdominal injuries [23]. However, there is a lack of clinical research on IL-17 and T-cell expression in patients with traumatic bowel rupture.

This prospective observational study aimed to investigate the serum levels of IL-17A in patients with traumatic bowel rupture and its clinical significance, focusing on the correlation of IL-17A with inflammatory factors and the relationship between IL-17A and preoperative severity and postoperative prognosis. This study may provide new comprehensive methods for predicting poor prognosis in patients with traumatic bowel rupture and offer new therapeutic targets for treatment.

## 2 Methods

### 2.1 Study population

This prospective observational study included 104 patients with traumatic bowel rupture admitted to Ruian People’s Hospital between June 2021 and June 2024. Patients were diagnosed with traumatic bowel rupture through abdominal puncture (significant free fluid in the abdomen) and imaging (CT showing free gas between the intestines and mesenteric edema). Inclusion criteria were as follows: (1) admission and treatment within 12 h post-trauma and (2) signed informed consent and follow-up. Exclusion criteria were as follows: (1) immune diseases; (2) pregnancy or lactation; (3) severe systemic diseases (e.g., severe heart, brain, liver, and kidney diseases); (4) traumatic brain injury; (5) severe infection before trauma; (6) malignant tumors; (7) bowel rupture due to other causes (e.g., bowel obstruction, intestinal tumors); and (8) severe damage to other organs. Patients were divided into mild/moderate bowel rupture (injury severity score [ISS] <16) and severe bowel rupture (ISS ≥16) groups based on their ISS scores upon admission.

### 2.2 Surgical treatment

All patients underwent standard surgical treatment for traumatic bowel rupture at our hospital. Mild bowel ruptures were treated with simple repair, while severe cases required partial bowel resection and anastomosis, with colostomy performed as needed. Abdominal cavity irrigation and drainage tubes were placed during surgery, and routine antibiotics were used postoperatively to prevent infection. All patients were followed up for 3 months post-discharge. Patients were divided into two groups based on follow-up outcomes: the good prognosis group (patients with well-healed wounds and no severe complications) and the poor prognosis group (patients with complications such as peritonitis, acute lung injury, sepsis, intestinal fistula, rebleeding requiring surgery, or death during follow-up).

### 2.3 Enzyme-linked immunosorbent assay (ELISA)

IL-17A is a pro-inflammatory cytokine that plays a crucial role in the immune response to injury and infection, particularly in the context of trauma [24]. Furthermore, IL-17A has been shown to interact with other cytokines such as IL-6, tumor necrosis factor-alpha (TNF-α), and C-reactive protein (CRP), which are known to be elevated in trauma patients [25,26]. Therefore, we chose to explore the relationship between IL-17A and inflammatory cytokines to better understand its potential as a biomarker for predicting prognosis in traumatic bowel rupture patients. Blood samples (5 mL) were collected from each patient’s elbow vein at 0, 24, 48, and 72 h and 7 days post-admission and placed in non-anticoagulant tubes. Serum samples were obtained by centrifugation at 3,500 rpm for 15 min. Serum IL-17A, IL-6, TNF-α, and CRP levels were measured using commercial ELISA kits (IL-17A no. MBS8123963, MyBioSource; IL-6 no. MBS8123859, MyBioSource; TNF-α no. MBS8123875, MyBioSource; CRP no. MBS8123937, MyBioSource) according to the manufacturer’s instructions.

### 2.4 Outcome measures

Demographic and clinical data were collected, including gender, age, body mass index, type of trauma (open or closed), cause of trauma (traffic accident, fall, assault, others), and trauma site (duodenum, cecum, small intestine, colon, rectum). Complete blood counts were performed at admission, and white blood cells (WBC), neutrophils (NE), and platelets (PLT) levels were recorded.

### 2.5 Statistical analysis

Statistical analysis was performed using SPSS 26.0. The normality of continuous data was tested using the Kolmogorov–Smirnov test. Normally distributed data were presented as mean ± standard deviation (SD), and non-normally distributed data as median (range). Comparisons between the two groups were made using the Student’s *t*-test or Mann–Whitney *U* test. Pearson correlation analysis was used for correlation analysis. The receiver operating characteristic (ROC) curve and area under the curve (AUC) were used to evaluate the diagnostic value of IL-17A for poor prognosis in traumatic bowel rupture. Logistic regression was used to analyze risk factors for poor prognosis, with poor prognosis as the dependent variable and related factors as independent variables. *p* < 0.05 was considered statistically significant.


**Informed consent:** All patients signed informed consent forms.
**Ethical approval:** This study was approved by the ethics committee of Ruian People’s Hospital, the ethics committee approval number No. RH-2021037.

## 3 Results

### 3.1 Baseline characteristics of all patients

This prospective study included a total of 104 patients with traumatic bowel rupture. The subjects were divided into two groups based on their ISS scores upon admission: ISS <16 group (61 patients) and ISS ≥16 group (43 patients). The clinical characteristics of all subjects are presented in Table 1. In the ISS ≥16 group, 55.8% of cases were due to traffic accidents, and 18.6% were due to falls. In the ISS <16 group, 50.8% of cases were due to traffic accidents, and 19.7% were due to falls. The ISS ≥16 group had significantly higher serum WBC and NE levels compared to the ISS <16 group (*p* < 0.05). No other significant differences were observed.

**Table 1:** Basic characteristics of all patients

Variable	ISS <16 group, *n* = 61	ISS ≥16 group, *n* = 43	*p*
Age (years)	48.30 ± 16.11	46.72 ± 13.41	0.601
Male, *n* (%)	46 (75.4)	33 (76.7)	0.829
BMI	23.31 ± 2.25	23.13 ± 2.18	0.439
Traffic accident, *n* (%)	31 (50.8)	24 (55.8)	0.479
Assault, *n* (%)	9 (14.5)	5 (11.6)	0.543
Fall, *n* (%)	12 (19.7)	8 (18.6)	0.843
Other, *n* (%)	9 (14.8)	6 (14.0)	0.872
Trauma site			
Colon, *n* (%)	16 (26.2)	11 (25.6)	0.923
Rectum, *n* (%)	7 (11.5)	5 (11.6)	0.982
Small intestine, *n* (%)	29 (47.5)	23 (53.5)	0.396
Other, *n* (%)	9 (14.8)	4 (9.3)	0.232
WBC (×10^9^/L)	8.10 ± 1.71	10.07 ± 1.98	<0.001
PLT (×10^9^/L)	186.35 ± 40.36	191.77 ± 40.71	0.504
NE (×10^9^/L)	4.66 ± 1.16	5.56 ± 1.40	0.001

BMI – body mass index, WBC – white blood cells, PLT – platelets, NE – neutrophils. Continuous data presented normal distribution were expressed by mean ± SD and analyzed by Student’s *t* test. Chi square test was used for rates.

### 3.2 Serum inflammatory cytokine levels in patients with traumatic bowel rupture

**Figure 1:**
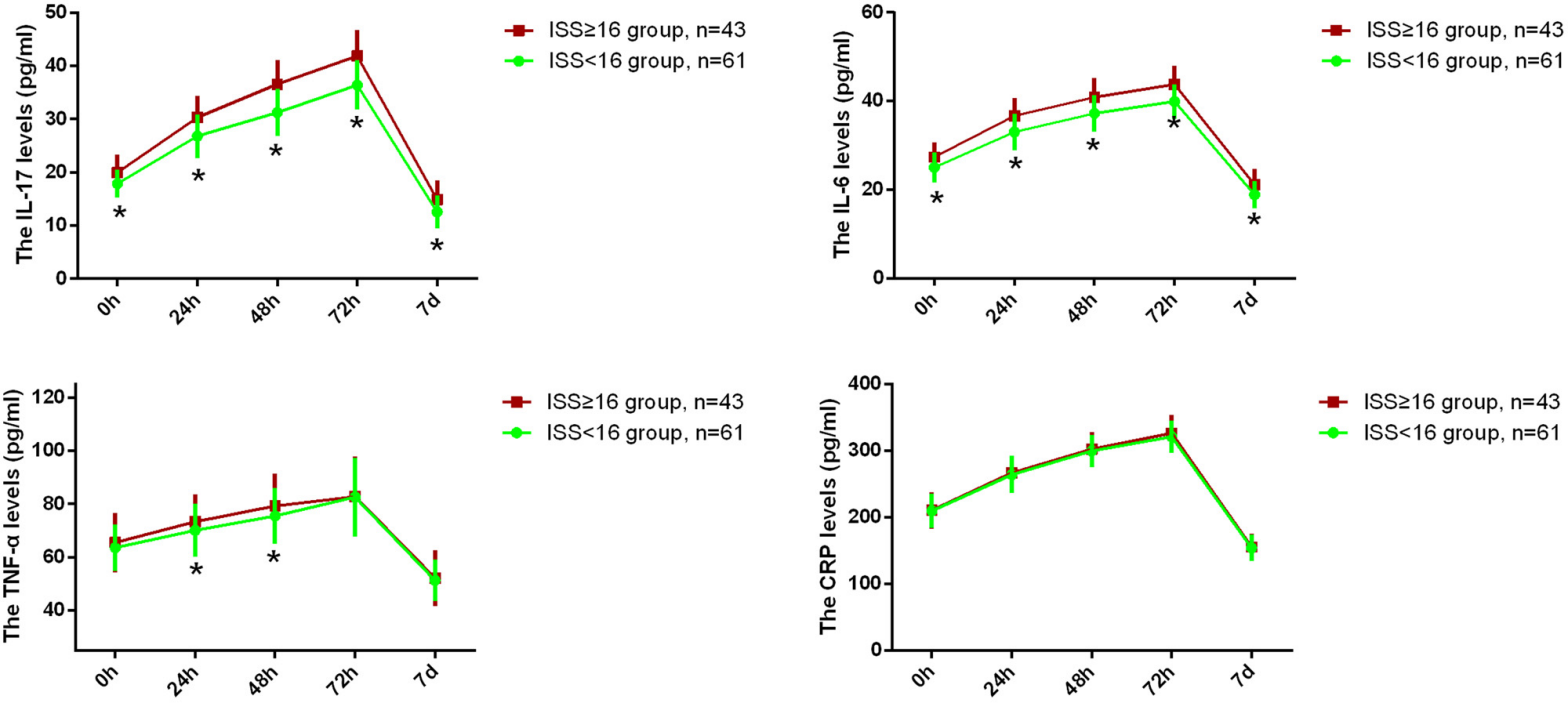
Serum levels of interleukin-17A (IL-17A), interleukin-6 (IL-6), tumor necrosis factor-alpha (TNF-α), and C-reactive protein (CRP) in patients with traumatic bowel rupture at 0 h (admission), 24, 48, and 72 h and 7 days post-surgery between ISS <16 group (green, *n* = 61) and ISS ≥16 group (red, *n* = 43). Student’s *t*-test for between-group comparisons. *p < 0.05.

We compared the serum levels of inflammatory cytokines IL-17A, IL-6, TNF-α, and CRP between the two groups at admission and various postoperative time points. As shown in Figure 1, the serum levels of IL-17A and IL-6 were significantly higher in the ISS ≥16 group compared to the ISS <16 group at admission. Additionally, serum levels of IL-17A, IL-6, TNF-α, and CRP gradually increased within 72 h post-surgery and then began to decrease. Moreover, we found that IL-17A and IL-6 levels remained significantly higher in the ISS ≥16 group at all time points compared to the ISS <16 group.

### 3.3 Relationship between serum IL-17A and prognosis in patients with traumatic bowel rupture

**Figure 2:**
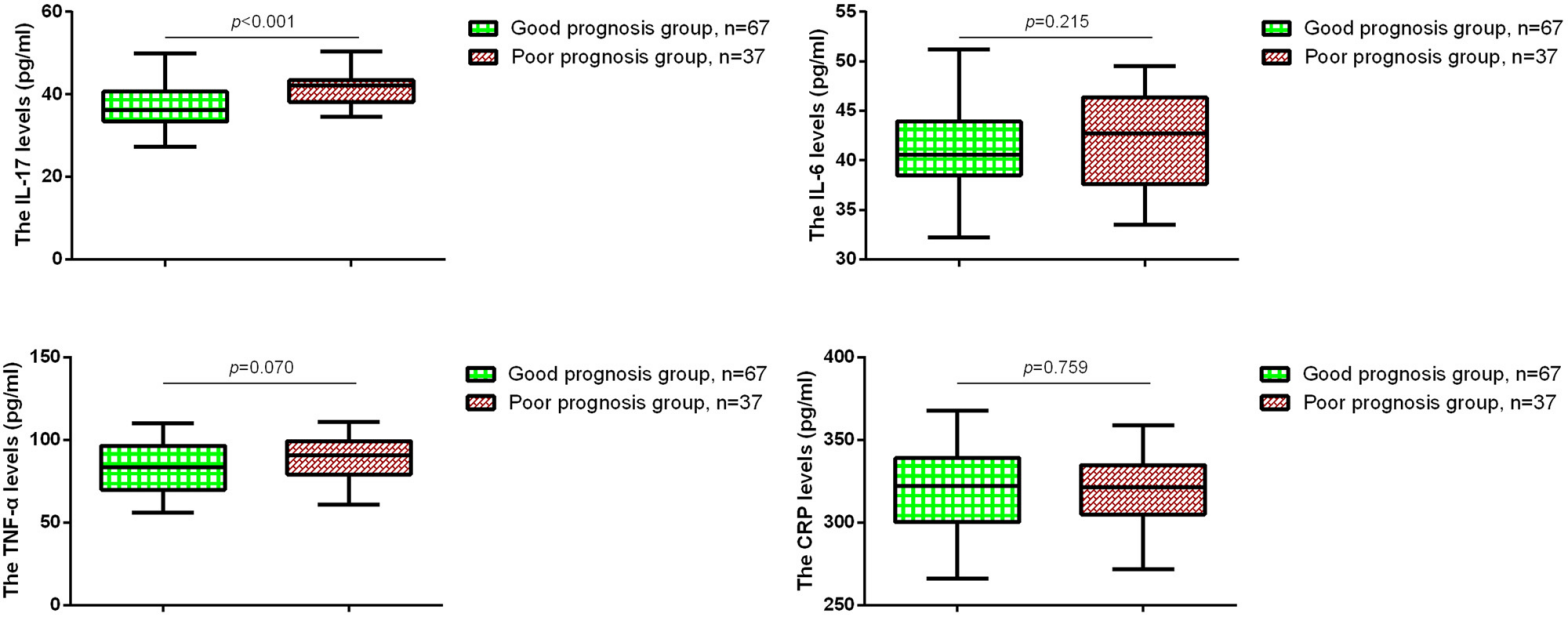
Serum levels of Interleukin-17A (IL-17A), interleukin-6 (IL-6), tumor necrosis factor-alpha (TNF-α), and C-reactive protein (CRP) in patients with traumatic bowel rupture at 72 h post-surgery between good prognosis group (*n* = 67) and poor prognosis group (*n* = 37). Student’s *t*-test for between-group comparisons.

Next, all patients were divided into two groups based on their prognosis: good prognosis group (*n* = 67) and poor prognosis group (*n* = 37). Since previous studies have shown that inflammatory cytokine levels peak at 72 h post-surgery, we compared the serum levels of IL-17A, IL-6, TNF-α, and CRP at 72 h post-surgery between the two groups. As shown in Figure 2, the poor prognosis group had significantly higher serum levels of IL-17A compared to the good prognosis group. No significant differences in serum IL-17A, IL-6, TNF-α, and CRP at 72 h post-surgery between traumatic bowel rupture of different sexes (Table 2).

**Table 2:** The serum inflammatory markers in patients with traumatic bowel rupture of different sexes

Variable	Male, *n* = 79	Female, *n* = 25	*p*
IL-17A (pg/mL)	38.15 ± 6.28	40.22 ± 6.27	0.096
IL-6 (pg/mL)	41.46 ± 4.45	41.66 ± 4.57	0.845
TNF-α (pg/mL)	86.10 ± 14.89	82.41 ± 14.40	0.278
CRP (pg/mL)	319.14 ± 26.02	319.31 ± 25.83	0.977

BMI – body mass index, WBC – white blood cells, PLT – platelets, NE – neutrophils, IL – interleukin, CRP – C-reactive protein, TNF-α – tumor necrosis factor-alpha. Continuous data presented normal distribution were expressed by mean ± SD and analyzed by Student’s *t* test.

### 3.4 Predictive value of serum IL-17A for poor prognosis in patients with traumatic bowel rupture

**Figure 3:**
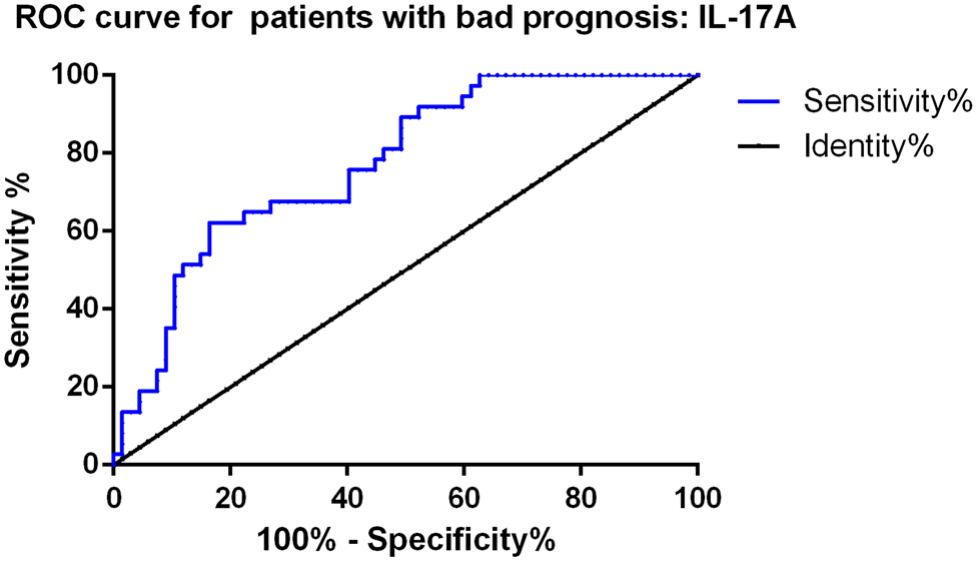
ROC curve analysis of serum IL-17A for poor prognosis in patients with traumatic bowel rupture. The AUC was 0.773, with a cutoff value of 41.75 pg/mL, a sensitivity of 62.2%, and a specificity of 83.6%. IL – interleukin.

We further analyzed the predictive value of serum IL-17A for poor prognosis in patients with traumatic bowel rupture using ROC curve analysis. As shown in Figure 3, serum IL-17A levels at 72 h post-surgery demonstrated a certain predictive value for poor prognosis, with an AUC of 0.773, a cutoff value of 41.75 pg/mL, a sensitivity of 62.2%, and a specificity of 83.6%.

### 3.5 Binary logistic regression analysis of risk factors for poor prognosis in patients with traumatic bowel rupture

Finally, to investigate the risk factors for poor prognosis in patients with traumatic bowel rupture, we conducted a multivariate binary logistic regression analysis. As shown in Table 3, the logistic regression analysis revealed that elevated levels of IL-17A at 72 h post-surgery were a significant risk factor for poor prognosis in these patients.

**Table 3:** Logistic regression analysis of risk factors for poor prognosis in patients with traumatic bowel rupture

Variables	Wald	Odds ratio	95% CI	*p*
Age	0.170	0.993	0.959–1.028	0.680
Sex	0.002	0.975	0.301–3.155	0.966
BMI	1.313	1.144	0.909–1.439	0.252
WBC	0.391	0.919	0.706–1.197	0.532
PLT	0.087	1.002	0.989–1.015	0.768
NE	1.383	1.262	0.856–1.861	0.240
Cause of accident	0.490	0.844	0.524–1.358	0.484
Trauma site	0.006	1.016	0.663–1.559	0.940
IL-6 at admission	0.528	0.944	0.809–1.103	0.468
CRP at admission	0.001	1.000	0.980–1.020	0.986
TNF-α at admission	0.112	1.008	0.960–1.059	0.738
IL-17 at admission	0.331	1.050	0.889–1.241	0.565
IL-6 at 72 h post-surgery	0.063	1.015	0.904–1.140	0.802
CRP at 72 h post-surgery	1.191	0.988	0.968–1.009	0.275
TNF-α at 72 h post-surgery	1.685	1.023	0.988–1.059	0.194
IL-17 at 72 h post-surgery	12.746	1.273	1.115–1.453	<0.001

BMI – body mass index, CI – confidence interval, WBC – white blood cells, PLT – platelets, NE – neutrophils, IL – interleukin, CRP – C-reactive protein, TNF-α – tumor necrosis factor-alpha.

## 4 Discussion

For the treatment of trauma, the timelier the intervention, the better. Accurate diagnosis is always a crucial component before formulating the correct treatment strategy. Patients with traumatic bowel rupture face higher infection risks due to the specific nature of their injuries, and the probability of postoperative complications is significantly increased. These complications not only severely threaten the patients’ life safety but also prolong hospitalization and increase psychological and economic burdens. Therefore, developing new biomarkers for the early diagnosis of the severity of traumatic bowel rupture and predicting the occurrence of postoperative complications is of great significance for timely and effective treatment interventions. In our study, we found that elevated serum IL-17A levels were associated with poor prognosis in patients with traumatic bowel rupture, highlighting the potential of IL-17A as a predictive biomarker. Moreover, the findings from the ROC curve analysis underscore the significant role of serum IL-17A as a predictive biomarker for poor prognosis in patients with traumatic bowel rupture.

An increasing number of biomarkers have been identified as clinically significant in the disease progression and prognosis of trauma patients. A clinical study found that serum levels of IL-6 and IL-8 were significantly elevated in trauma patients, serving as reliable markers of immune response in these patients [27]. Park and Hwang found that serum levels of S100 calcium-binding protein β (S100-β) and neuron-specific enolase (NSE) in patients with brain injuries were associated with initial Glasgow Coma Scale (GCS) scores and clinical outcomes. The levels of S100-β and NSE gradually decreased during subsequent treatment and follow-up, correlating with GCS scores 6 months later, indicating that these biomarkers could help evaluate patient condition and clinical outcomes [28]. Sun and Xia conducted a clinical study involving 299 patients with abdominal trauma, revealing that serum levels of NOD-like receptor family, pyrin domain containing 3 (NLRP3), and high mobility group box 1 (HMGB-1) were elevated in these patients. The study found that these elevated levels were associated with a higher 6-month mortality rate in patients with severe abdominal trauma. NLRP3 and HMGB-1 were identified as potential new therapeutic targets for treating patients with severe abdominal trauma [29]. Similarly, research by Sousa et al. demonstrated that pro-inflammatory and anti-inflammatory responses occur simultaneously in the early stages of trauma. They found that measuring cytokine levels, particularly IL-10 within 48–72 h post-trauma, could help assess outcomes. In the near future, modulating these cytokines may become a new option for treating severe trauma [30]. In our study, we similarly found that elevated IL-6 and IL-17A levels were correlated with the severity and prognosis of traumatic bowel rupture, reinforcing its potential role as a biomarker for clinical assessment.

IL-17 has been highlighted as a biomarker associated with inflammation, infection, trauma, and various disease diagnoses and prognoses in numerous molecular and clinical studies [31]. Al-Saadany et al. found significantly elevated serum IL-17 levels in patients with rheumatoid arthritis, with higher levels during active disease compared to stable phases, indicating its role in regulating inflammation and pathogenesis in these patients [32]. In a rat model of multiple trauma, Dai et al. discovered that immune imbalance plays a role in the pathogenesis and progression of multiple trauma, and changes in IL-17 levels might predict therapeutic responses and clinical progression [33]. Studies have also reported the association of IL-17 with gastrointestinal diseases. Rampal et al. found upregulated IL-17 expression in patients with ulcerative colitis, with all-trans retinoic acid (RA) playing a key role in promoting intestinal inflammation by upregulating IL-17 [34]. Song et al. established a mouse burn model and found that the intestinal mucosal barrier was disrupted after burns, with IL-17 neutralization protecting the intestinal mucosal barrier by affecting the expression of pro-inflammatory cytokines [35]. Their study suggested that IL-17 blockade could provide a unique target for therapeutic intervention in burn-induced intestinal injuries.

In our study, we found that elevated serum IL-17A levels were associated with poor prognosis in patients with traumatic bowel rupture, highlighting the potential of IL-17A as a predictive biomarker. IL-17A is a key pro-inflammatory cytokine that modulates immune responses, particularly in acute inflammation and trauma [36]. IL-17A contributes to the recruitment of neutrophils and the amplification of inflammatory cascades, which are critical in the context of trauma-related injuries [37]. Hefele et al. demonstrated that trauma induces increased IL-17A expression on Th17 and CD4+ Treg cells [24]. Similarly, Ahmed Ali et al. found that elevated serum IL-17 levels were strongly associated with increased susceptibility to sepsis in major trauma patients [38]. These findings underscore IL-17A’s importance in acute and traumatic settings, supporting its use as a prognostic biomarker in patients with traumatic bowel rupture.

Additionally, the findings from the ROC curve analysis underscore the significant role of serum IL-17A as a predictive biomarker for poor prognosis in patients with traumatic bowel rupture. With an AUC of 0.773, IL-17A was shown to have a moderate but reliable capacity to distinguish between patients with good and poor outcomes, which is a crucial step towards improving patient prognosis assessment. The sensitivity of 62.2% and the specificity of 83.6% suggest that serum IL-17A levels could serve as a valuable tool in clinical practice for identifying high-risk patients early. The identified threshold of 41.75 pg/mL provides clinicians with a measurable parameter to stratify patients based on their risk of poor outcomes. By incorporating IL-17A measurements into routine monitoring, clinicians could prioritize early interventions for high-risk patients, potentially improving management and outcomes. Furthermore, the logistic regression analysis supports elevated IL-17A levels as an independent risk factor for poor prognosis, reinforcing its potential as a cornerstone in future prognostic models, especially when combined with other clinical indicators to enhance predictive accuracy.

This study has several limitations. First, the sample size is relatively small, which may limit the generalizability of our findings. Second, the study was conducted at a single center, and multicenter studies are needed to validate our results. Third, the mechanisms underlying the elevation of IL-17A in traumatic bowel rupture patients were not investigated, warranting further research to understand the pathophysiological role of IL-17A in this context. Finally, while the ROC analysis showed that IL-17A at 72 h post-operation has moderate predictive value, its sensitivity of 62.2% indicates that a considerable portion of poor outcomes might be missed when IL-17A is used alone. Future studies should explore the potential of combining IL-17A with other biomarkers to improve predictive accuracy and clinical applicability.

## 5 Conclusion

In summary, timely and accurate diagnosis is essential for the effective treatment of trauma, especially in cases of traumatic bowel rupture. Our study highlights the significant potential of serum IL-17A as a reliable and predictive biomarker for predicting poor prognosis in these patients. Moreover, elevated IL-17A levels may not only serve as a prognostic biomarker but could also represent a potential therapeutic target for modulating the inflammatory response in trauma patients. Targeting IL-17A or its associated pathways could offer new avenues for therapeutic interventions aimed at reducing inflammation and improving patient outcomes in traumatic injuries.
